# Experience of depression: Need for support

**DOI:** 10.4103/0019-5545.43068

**Published:** 2005

**Authors:** Pratibha Bajpai

I must say at the outset that the year 2005 has been the worst year of my life, mainly because this year I was declared a patient of depression. My broken marriage, loneliness, underemployment…these surely have been some of the reasons. I am a very introverted person. It is not easy for me to open up and talk about how I feel. For someone who hates to admit defeat in life, my marriage was a major defeat. I was married for five years. It was an arranged marriage but it was more like a bad decision that affects one's whole life. Everything was going in the wrong direction. That life had nothing I could have identified with. I don't blame anybody.

Divorce is never smooth but it was over and done with. I came to Mumbai from Kanpur. I worked for a couple of newspapers, one publication house, got involved in theatre activities. Life was once again busy and creative. I think I managed a good show for ten long years! My parents are now old and far away. They do not influence my life anymore but my brother has been a pillar of strength. He would never let me give up. In Mumbai I stayed at his place and was accepted by his family. But I could never forget that it was not my home. I was very vulnerable to any imagined hurt and was always insecure. The stress I felt was real and palpable. At times, I felt choked with emotions—I couldn't breathe, couldn't speak properly. I did not suspect that months and months of sleepless nights was not something normal. I used to cry a lot. When a person cries a lot, it is a symptom. If she is crying without sufficient reason, cries over apparently small matters, loses her temper often—we should definitely look deep inside the person. I can say that today, but I did not take my grief or my discontent seriously enough for a very long time.

I work in creative fields which have no easy ‘routines’. No guarantees either. To be able to survive on your own terms is a success but that is not something one can hold on to and show to the world. Besides, I am 41 now. The physical stamina is diminishing. I cannot be the same young enthusiastic journalist forever. My work has kept me busy but it cannot replace emotional fulfilment. I have never attempted suicide, but sometimes when I ask myself why am I living—nobody needs me, my death may not cause a single ripple in this world—all my confidence vanishes and life loses its meaning and purpose. Remarrying could have been a solution but I did not meet anyone. And now, I have got used to living alone and may not accept anybody's interference. So the vacuum remains.

This was the background in which I was trying to concentrate on my new assignment. Then I started having headaches which became severe—so bad that despite painkillers, the pain was impossible to bear. I was just not able to work. The dreadful routine of sleepless nights returned. I went to Kanpur, to my parents' place. I met our family doctor there and was asked to get certain tests done. Except for high blood pressure, all the tests were normal. Then I was referred to the psychiatrist and he said I was in severe depression.

The diagnosis surprised me. I liked to think of myself as a confident and strong person who could face anything. It was not easy to accept this diagnosis. It was as if all my desperate efforts, creative assignments, hard-earned peace of mind and those ten years in between had meant nothing.

‘Depression! But she does not look like having depression. She is talking quite logically.’ That was the reaction at home. Anyway, the treatment started and it was a nightmare! My life was completely shattered. The headaches and sleepless nights continued. With the treatment, I developed constipation and then piles, which used to bleed. There was pain in the legs. I was scared to eat. I became physically so weak that I needed support to go to the toilet. I felt dazed all the time. I had to be attended to like a child. My old parents used to wake up several times at night to comfort me during my helpless moanings.

In Kanpur, no counselling was offered to me. The approach was entirely clinical and cold. If I complained of ‘side-effects’, I was told ‘It would be all right, just keep taking tablets!’ If I still complained, instead of some help, they asked me to get admitted. Once I was jeeringly told, ‘There are many stray pigs in Kanpur, why don't you do an article on them?’ I was so angry. Had I been in a state to do articles, I would not have gone to them. My parents and I were going through hell. I still do not say the treatment was useless or anything like that. But the doctors lacked sympathy.

After those four unending months in Kanpur, I came to Mumbai for treatment. Here they said that my dosages were too heavy. All the tablets were changed and the dosages adjusted. The treatment here is friendly. I feel okay. I still take some ten tablets a day. The body cycle is so disrupted that nothing is in order yet. My head and the whole body feel heavy. I cannot go out as much as I would like to. When on the road, I have to force myself to remain conscious or I get lost. I do not tell this to people at home. To make a simple phone call, I have to muster courage. I feel scared that I would say something wrong. Still, I did two programmes for the All India Radio and I am presently working on a book. I must mention that I have had tremendous family support. My brother forces me to live. I dread to think of those like me who are suffering without love and support.

My grief has taught me certain valuable lessons; that life is not black and white. There is much between the two. My life has made me intense. I have discovered that I cannot stand general chatter any more. I feel very restless on such occasions. I search for meaning in everything. All of us live. Sometimes we become sad. But not everyone touches the bottom to feel the depth of the grief. Nobody understands him or her and nobody wants to understand either. Even within families, the communication gap is increasing. The blood relatives do not want to get involved beyond a point. Someone who cries too much is a liability; someone who has lost interest in life is called lazy; someone who is hurt too much and vents his anger is an uncivilized beast. People have ready labels by which they classify and discard.

So what happens to them who are thus marginalized by society? During the period of my suffering, and as I recover, I have tried reflecting on this issue.

- When I try to analyse as a journalist, I find the supportive mental health structure very inadequate for our society which is still feudal in its relationships. Around me I find all women with different levels of depression, which is never diagnosed, hence never treated. Crying and cribbing, slogging for their entire lives and dying in discontent, is quite normal for women in our society. For generations, our families have learnt to effectively ignore women's grief. The desensitization towards women's complaints is so total that women themselves would not identify these as problems. Instead they call it women's destiny. If someone like me rebels, she gets isolated, and at some point in life gets tired fighting. There is a real gap between our women and the ‘new, politically correct, gender-sensitive’ research in the field of mental health. I do not see how we can bridge this gap.
- I was treated by private practitioners. Their counselling fees and the newer, safe tablets were certainly not cheap. Still, I was not taken to a general hospital. Why? One reason is, I belong to the middle class and this class does not trust government hospitals. But another may be that such traumas are zealously guarded as ‘private’. So to make our huge, remote psychiatry wards respond to the subtleties around remains a challenge. If the depressed do not reach hospitals, can the hospitals reach them instead?
- In Kanpur and later in Mumbai, I enquired if there is any helpline for patients with depression. It does not exist. I feel patients with depression should have support groups such as ‘Alcoholics Anonymous’. Some network, through which they may be able to help each other. They can understand how it hurts when someone is sinking to that bottom. They have the compassion. In the middle of the night when your head is bursting with all the injustice, the grief, the hopelessness of your situation and real, physical pain, you need and deserve the help which professionals cannot render and your exhausted relatives cannot offer. Just a phone call and an objective, yet sympathetic voice on the other end, which can help with simple suggestions such as ‘Okay, you try a hot water bath and may be spread a clean, white bedsheet on your bed’—makes a world of difference. If it is an emergency, the helpline could coordinate to provide professional help through functioning, community-based networks. It is workable. I have thought about this several times.

I am sure, patients like me would consider this a very meaningful work. I am interested in these efforts because I hope it may fill my own vacuum. I am now looking for possibilities through which we can make this happen. Because my grief has a productive role in helping others. Our suffering was not in vain. We can reduce each other's suffering.

